# Being overweight and obese increases suicide risk, the severity of depression, and the inflammatory response in adolescents with major depressive disorders

**DOI:** 10.3389/fimmu.2023.1197775

**Published:** 2023-11-01

**Authors:** Putrada Ninla-aesong, Pavarud Puangsri, Pongtorn Kietdumrongwong, Haruthai Jongkrijak, Kusumarn Noipha

**Affiliations:** ^1^ Department of Medical Science, School of Medicine, Walailak University, Nakhon Si Thammarat, Thailand; ^2^ Research Center in Tropical Pathobiology, Walailak University, Nakhon Si Thammarat, Thailand; ^3^ Excellence Center of Community Health Promotion, Walailak University, Nakhon Si Thammarat, Thailand; ^4^ Department of Medical Clinical Science, School of Medicine, Walailak University, Nakhon Si Thammarat, Thailand; ^5^ BDMS Health Research Center, Bangkok Dusit Medical Service PLC, Bangkok, Thailand; ^6^ Walailak University Hospital, Walailak University, Nakhon Si Thammarat, Thailand; ^7^ Faculty of Health and Sports Science, Thaksin University, Paphayom, Phatthalung, Thailand

**Keywords:** depression, inflammation, inflammatory markers, overweight, obese, IL-1b, IL-6, suicide

## Abstract

**Background:**

There is a bidirectional relationship between obesity and depression. We investigated whether the coexistence of obesity and depression increases the risk of having severe depression and a high suicide risk in adolescents with major depressive disorder (MDD). Additionally, we explored the potential mechanisms linking the coexistence of obesity and depression to worse outcomes in these patients.

**Methods:**

The odds of high suicide risk and severe depression were compared among MDD patients based on different body mass index (BMI) groups. Complete blood count (CBC) parameters, inflammatory ratios (neutrophil–lymphocyte ratio [NLR], monocyte-lymphocyte ratio [MLR], and platelet-lymphocyte ratio [PLR]), and cytokine levels (IFN-γ, IL-1β, IL-6, IL-8, MCP-1, TNF-α, and TGF-β1) were evaluated across BMI groups. Additionally, Pearson correlation coefficients (*r*) were assessed to understand the relationships between the 8Q and 9Q scores, CBC parameters, inflammatory ratios, cytokine levels, and BMI.

**Results:**

A total of 135 antidepressant-naive adolescents with MDD were included. Overweight and obese MDD patients had higher odds of having high suicide risk and severe depression than lean individuals. Furthermore, they exhibited significantly higher white blood cell (WBC), and neutrophil counts. The NLR tended to be higher in obese MDD patients than in leans. Overweight and obese MDD patients had elevated levels of interleukin (IL)-1β and IL-6 compared to lean individuals, while TGF-β1 levels appeared to decline as body weight increased. BMI showed weak positive correlations with 8Q score, WBC count, neutrophil count, monocyte count, platelet count, neutrophil percentage, and NLR, and a weak negative correlation with lymphocyte percentage. The 8Q score displayed weak positive correlations with BMI, neutrophil percentage, monocyte percentages, NLR, and MLR, and a weak negative correlation with lymphocyte percentage.

**Conclusion:**

The findings suggest that coexistence of overweight or obesity with depression heightened inflammatory responses, leading to worse outcomes and increased suicide risk in adolescents MDD patients.

## 1 Introduction

Depression is a common mental health disorders, affecting approximately 5% of adults worldwide. It is the second leading cause of death among people aged 15–29 (1). This condition is characterized as a chronic low-grade systemic inflammatory disease (2–4). Evidence from recent studies supports the notion that inflammatory processes play a crucial role in the pathophysiology of depression (3, 5–9). Various factors seem to be related to systemic inflammation and an increased risk of developing depression. These factors include stress, obesity, smoking, lack of physical activity, sleep disturbances, unhealthy diet, immune system dysregulation, cytokine imbalances, hormonal factors, increased gut permeability, and medication and medical conditions (10).

Obesity poses a significant health concern. In 2016, there were 1.9 billion overweight adults globally, of which 650 million were classified as obese. It is estimated that the number of individuals suffering from obesity worldwide will reach one billion by the year 2030 (10). Obesity and overweight are associated with an increased risk of various health conditions and diseases. These conditions can range from cardiovascular diseases to metabolic disorders (4, 11–16). These include cardiovascular diseases, type 2 diabetes, dyslipidemia, hypertension, certain cancers, sleep apnea and respiratory disorders, musculoskeletal conditions like osteoarthritis, susceptibility to infectious diseases, and even mental health disorders such as depression, and related conditions (10–16). If this trend continues, obesity-related conditions will likely become more prevalent. According to the global burden of disease, over 4 million people die each year as a result of being overweight or obese. Recently, studies have shown that obesity affects immune function and increases the risk of severe illness from coronavirus disease of 2019 (COVID-19) infection (17, 18).

White or visceral adipose tissue is the main depot for storing fat and serves as the largest endocrine organ that secretes adipokines, growth factors, cytokines, and chemokines into the bloodstream that affect endocrine and immune functions (2, 13, 15, 16, 18–23). Weight gain, obesity, chronic overnutrition, and aging lead to alterations in the white adipose tissue phenotype, characterized by the appearance of inflamed, dysfunctional, along with an increased infiltration of immune cells such as macrophages, T cells, and eosinophils (13). This inflammatory milieu leads to the release of proinflammatory cytokines and chemokines like interleukin-6 (IL-6), tumor necrosis factor-alpha (TNF-α), and monocyte chemoattractant protein-1 (MCP-1) (8, 13, 15, 20, 22, 23). Such alterations trigger uncontrolled inflammatory responses, resulting in systemic low-grade inflammation. Consequently, chronic low-grade systemic inflammation of adipose tissue contributes to insulin resistance, a hallmark of obesity-related metabolic stress and the development of metabolic diseases (2, 4, 11–16). Previous studies have highlighted the link between obesity or overweight conditions and uncontrolled inflammatory responses or immune dysfunctions (2, 4, 11–16, 18–21).

Three systematic reviews and meta-analyses have illustrated the bi-directional relationship between obesity and depression (24–26). Firstly, Luppino et al. (2010) (24) conducted a systematic review and meta-analysis of 15 longitudinal studies. They found that overweight or obesity increased the risk of depression among adults (aged 20 years or older) but not among younger individuals (age < 20 years). Moreover, depression was found to increase the odds of developing obesity. In a subsequent systematic review and meta-analysis of 19 studies, Mannan et al. (2016) (25) confirmed this bi-directional link between obesity and depression. Their findings suggest that the direction of depression leading to obesity is stronger than the reverse direction. Furthermore, women in their reproductive years (18-49 years) are more susceptible to developing depression, compared with women more than 49 years old. A recent systematic review and meta-analysis study by Blasco et al. (2020) (26), covering 18 studies (9 cross-sectional, 6 longitudinal, and 3 clinical trials), also confirmed the bidirectional relationship between depression and obesity. It highlighted that obesity increases the risk of depression, particularly in women with recurrent depressive disorder, while depression, in turn, becomes a risk factor for obesity. Additionally, when obesity and depression coexist, it often results in poorer illness outcomes. Specifically, MDD patients with the highest BMI category reported more medical and psychiatric comorbidities. However, the precise mechanisms explaining how obesity leads to its complications, and those that explain how the coexistence of obesity and depression impacts the poor prognosis of depression remain unknown.

Neutrophils, monocytes, lymphocytes, and platelets serve as sources of substances involved in regulating inflammation, which are relevant to the pathophysiology of depression and contribute to depressive symptoms (3, 6–8). Neutrophil–lymphocyte ratio (NLR), monocyte-lymphocyte ratio (MLR), and platelet-lymphocyte ratio (PLR) are considered biomarkers of systemic inflammation. Recently, inflammatory ratios have been spotlighted as potential simple biomarkers for depression. Notably, a higher NLR has been linked to greater depression severity and has been proposed as a biomarker of suicidal risk in individuals with depression (27–30). Similarly, higher MLR, PLR, and platelet count are associated with more severe depression and an elevated risk of suicide (31–33). An increased MLR has been linked to both depression and suicidal severity in depressed patients (31, 34). Furthermore, a higher MLR has been suggested to be a more predictive biomarker for suicide attempts in adolescents with MDD than either NLR or PLR (31). Furthermore, MLR appears to be more responsive to selective serotonin reuptake inhibitor (SSRI) treatment compared to NLR or PLR (32). Additionally, higher baseline platelet counts have been associated with non-response to SSRI treatment in adolescents with MDD (32).

This study aimed to investigate whether the coexistence of obesity and depression increases the risk of having severe depression and a high suicide risk in adolescents with MDD. Additionally, we explored the potential mechanisms that link the comorbidity of obesity and depression to the poor prognosis in these adolescents, focusing on changes in complete blood count (CBC) parameters, inflammatory ratios (NLR, MLR, and PLR), and cytokine levels due to obesity. To achieve this, the odds of having high suicide risk and severe depression in patients with MDD were compared among different BMI groups. Furthermore, we compared CBC parameters, inflammatory ratios, and levels of proinflammatory cytokines (interferon (IFN)-γ, IL-1β, IL-6, IL-8, MCP-1, and TNF-α) and anti-inflammatory cytokine (transforming growth factor-beta 1 [TGF-β1]) between the different BMI groups. Lastly, we investigated the Pearson correlation coefficients (*r*) between the 8Q and 9Q scores, CBC parameters, and inflammatory ratios with BMI.

## 2 Materials and methods

### 2.1 Study design and participants

This study employed a cross-sectional design, involving university students aged 18–24 years who were visiting the psychiatry clinic, the outpatient department, of Walailak University Hospital in Nakhon Si Thammarat, southern Thailand. This study was conducted between November 2020 and March 2021. It was approved by The Institutional Review Board of Walailak University (WU-EC-MD-1-111-66), and all participants provided written informed consent. Each participant was diagnosed with MDD and had not been previously exposed to any antidepressant treatment. All adolescents diagnosed with MDD who were naïve to antidepressants and met the inclusion criteria were invited to participate in the study.

### 2.2 Diagnosis of major depressive disorder

A licensed psychiatrist made the diagnosis based on the criteria of the American Psychiatric Association’s Diagnostic and Statistical Manual of Mental Disorders, Fifth Edition (DSM-V) (31, 32).

### 2.3 Inclusion and exclusion criteria

All participants underwent comprehensive evaluation that included a routine physical examination, an assessment of physical health comorbidities, screening for COVID-19 infection using antigen test kit, an evaluation of other mental illnesses, examination of additional mental health data, and a review of their medical histories. A complete blood count (CBC) with differential analysis was performed on each participant. Additional laboratory tests and investigations were conducted if there were any concerns regarding physical health issues (31, 32).

#### 2.3.1 Inclusion criteria

All patients diagnosed with MDD, aged 18–24 years, who were antidepressant-naive and visiting the outpatient unit of the university hospital, were included in the study when they provided informed consent and did not meet the specific exclusion criteria.

#### 2.3.2 Exclusion criteria

The participants were excluded from the study if they met any of the following conditions: 1) physical comorbidities, except for being overweight or obese; 2) presence of fever, infectious diseases, or recent infections; 3) COVID-19 infection; 4) chronic physical illnesses or inflammatory diseases; 5) use of anti-inflammatory or immunosuppressive drugs; 6) other mood disorders or mental illnesses; 7) immune system and hematological system disorders; 8) pregnancy or having been pregnant within the past six months; and 9) usage of substances and alcohol or smoking more than 10 cigarettes per day (31, 32).

### 2.4 Severity of depression, suicidal behavior, and suicidality severity

The Patient Health Questionnaire-9 (PHQ-9 or 9Q) scale, a depression rating scale, was employed to evaluate the severity of depression. The scores of 5, 10, 15, and 20 on the PHQ-9 (9Q) scale correspond to mild, moderate, moderately severe, and severe depression, respectively. The assessment of suicidal behavior and suicidality severity were conducted using the 8Q scale, a Thai version of a suicidality module of the Mini International Neuropsychiatric Interview (M.I.N.I)5.0.0 (35). Based on the 8Q scale, suicidality was classified into four levels: no suicide risk (score = 0), low suicide risk (score = 1–8), moderate suicide risk (score = 9–16), and high suicide risk (score ≥ 17). Furthermore, participants were divided into three classification based on their suicidal behaviors:1) those without suicidal ideation (NSI), 2) those with suicidal ideation (SI), and 3) those who had attempted suicide (SA) (31, 32).

### 2.5 BMI measurement

Body mass index (BMI) was calculated using the formula of weight in kilograms divided by the height in meters squared (kg/m^2^). The BMI classification were as follow: A BMI of less than 24.9 kg/m^2^ was considered as lean, a BMI ranging from 25.0 to 29.9 kg/m^2^ was considered as overweight, and a BMI of 30 kg/m^2^ or higher was obese.

### 2.6 Laboratory study

Prior to beginning MDD-specific treatment, a 10-mL blood sample was collected from each participant. The complete blood count (CBC) with differential analysis required 2–3 mL. The remaining 7–8 mL were allowed to clot and subsequently utilized for the measurement of cytokines through the enzyme-linked immunosorbent assay (ELISA) technique.

#### 2.6.1 Complete blood count test and inflammatory ratios

A routine CBC with differential analysis was conducted using an automated analyzer at the Central Laboratory of Walailak University Hospital. The inflammatory ratios, including NLR, PLR, and MLR, were calculated from the CBC parameters.

#### 2.6.2 Cytokine measurement by ELISA

Sera were extracted from clotted blood and stored at -80°C until ready for analysis. The cytokines, including IFN-γ, IL-1β, IL-6, IL-8, MCP-1, TNF-α, and TGF-β1, were analyzed from serum samples using commercial ELISA kits as per the manufacturer’s instructions. The ELISA kits for measuring IFN-γ, IL-1β, IL-6, IL-8, and MCP-1 were purchased from BD Biosciences, while those for IL-1β, TNF-α, and TGF-β1 were purchased from R & D Systems. All samples were measured in duplicate (36).

### 2.7 Statistic analysis

Statistical analyses were performed with SPSS software (version 17.0, Chicago, IL, USA), except for the calculation of odds ratio, which was computed using an online calculator (37). To compare the groups, the chi-square test and analysis of variance (ANOVA) were used. The results are presented as mean ± SEM, SD, or odds ratios. The linear relationship between two continuous variables was conveyed through the Pearson correlation coefficient (*r*). A p-value less than 0.05 was considered statistically significant.

## 3 Results

The characteristics of the study participants are detailed in **Supplementary Table S1**. This study included 135 antidepressant-naïve adolescents diagnosed with MDD. Among them, 72.6% (98 participants) were lean, 14.1% (19 participants) were overweight, and 13.3% (18 participants) were obese MDD participants. Furthermore, 69.6% of the participants were female, and 91.1% were experiencing their first depressive episode. No significant differences in age, state of depressive episode status, and smoking status were observed between groups (**Supplementary Table S1**).

### 3.1 Association between 8Q and 9Q scores, suicide risk, depressive severity, suicidal behavior, and BMI in patients with MDD

The 8Q and 9Q scores, suicide risk, depressive severity, and suicidal behavior in patients with MDD with different BMI groups are shown in **Supplementary Table S2**. Overweight and obese patients with MDD were more likely to have higher 8Q and 9Q scores, a higher suicide risk, severe depression, and attempted suicide than lean patients with MDD, but this was not statistically significant (**Supplementary Table S2**).

**Table 1** shows the impact of being overweight or obese on the odds of having high suicide risk and severe depression in patients with MDD. Overweight and obese individuals had higher odds of having high suicide risk (odds ratios: overweight, 3.02; 95% CI, 0.98–9.34, p -value of 0.028; obese, 2.51; 95% CI, 0.77–8.23, p -value of 0.063; overweight or obese, 2.77; 95% CI, 1.11–6.91, p -value of 0.015) and severe depression compared to lean individuals (odds ratios: overweight, 3.11; 95% CI, 1.12–8.60, p -value of 0.014; obese, 1.73; 95% CI, 0.58–5.13, *p-*value of 0.163; overweight or obese, 2.36; 95% CI, 1.05–5.29, p = 0.019).

**Table 1:** Influence of being overweight or obese on odds of having high suicide risk and severe depression in patients with MDD.

	High suicide risk					Severe depression				
	Yes (*n*)	No (*n*)	OR	95% CI	P-value	Yes (*n*)	No (*n*)	OR	95% CI	P-value
Overweight	6	13	3.02	0.98 - 9.34	0.028*	9	10	3.11	1.12 - 8.60	0.014*
Obese	5	13	2.51	0.77- 8.23	0.063	6	12	1.73	0.58 - 5.13	0.163
Overweight or obese	11	26	2.77	1.11- 6.91	0.015*	15	22	2.36	1.05 - 5.29	0.019*
Lean	13	85				22	76			

Data are shown as Odds ratio. Analyses were performed using Fisher’s Exact test. * Statistical significance was set at p < 0.05.

Odds ratio, exposure = overweight or obese MDD patients, control = lean MDD patients. Based on the 8Q score, suicidality can be classified as high suicide risk when 8Q score ≥ 17. PHQ-9 scores of ≥ 20 represent severe depression. * Statistical significance was set at p < 0.05.

MDD, major depressive disorder; n, events; OR, odds ratio; 95% CI, 95% confidence intervals.

### 3.2 The complete blood count parameters and inflammatory ratios in different BMI groups in patients with MDD

**Table 2** summarizes the CBC parameters and inflammatory ratios in patients with MDD in different BMI groups. One-way ANOVA revealed significant differences in red blood cell (RBC) count and hematocrit (p = 0.024 and 0.044, respectively). WBC and neutrophil counts increased significantly with increasing body weight in patients with MDD (p = < 0.0001 and < 0.0001, respectively). The lymphocyte percentage decreased with increasing body weight in patients with MDD, but this was not statistically different. Platelet count and NLR increased with increasing body weight in patients with MDD, but this was not statistically significant. Hemoglobin and monocyte counts were higher in overweight and obese patients with MDD compared to lean patients with MDD, but the difference was not statistically significant (p = 0.062 and 0.066, respectively).

**Table 2:** The complete blood count parameters and inflammatory ratios in patients with MDD with different BMI groups.

Parameters	All MDD (*n*=135)	Lean MDD (*n* =98)	Overweight MDD (*n* =19)	Obese MDD (*n* =18)	P -value
RBC count (x10^6^ cells/mm^3^)	4.85 ± 0.05	4.78 ± 0.05^a^	5.10 ± 0.14	5.00 ± 0.12	0.024*
Hemoglobin (g/dL)	13.24 ± 0.13	13.05 ± 0.16	13.86 ± 0.36	13.64 ± 0.31	0.062
Hematocrit (%)	41.00 ± 0.36	40.45 ± 0.42^a^	42.61 ± 0.95	42.29 ± 0.88	0.044*
MCV (fl)	85.15 ± 0.64	85.23 ± 0.79	84.58 ± 1.36	85.28 ± 1.74	0.939
MCH (pg)	27.45 ± 0.26	27.47 ± 0.33	27.2 ± 0.59	27.47 ± 0.66	0.966
MCHC (%)	32.17 ± 0.09	32.17 ± 0.11	32.20 ± 0.23	32.11 ± 0.17	0.960
RDW (%)	13.19 ± 0.13	13.22 ± 0.17	13.19 ± 0.19	13.03 ± 0.29	0.886
WBC count (x10^3^cells/mm^3^)	7.91 ± 0.19	7.42 ± 0.20^a,b^	9.05 ± 0.44	9.41 ± 0.51	<0.0001*
Neutrophil count (x10^3^ cells/mm^3^)	4.82 ± 0.13	4.48 ± 0.14^a,b^	5.54 ± 0.36	5.91 ± 0.40	<0.0001*
Neutrophil (%)	60.53 ± 0.52	60.18 ± 0.59	60.63 ± 1.33	62.33 ± 1.82	0.389
Lymphocyte count (x10^3^ cells/mm^3^)	2.51 ± 0.06	2.39 ± 0.07 ^a,b^	2.82 ± 0.12	2.82 ± 0.20	0.008*
Lymphocyte (%)	32.02 ± 0.47	32.43 ± 0.55	31.68 ± 1.10	30.17 ± 1.58	0.271
Monocyte count (cells/mm^3^)	411.4 ± 19.5	384.1 ± 21.0	500.7 ± 53.9	466.0 ± 67.9	0.066
Monocyte (%)	5.33 ± 0.24	5.32 ± 0.28	5.68 ± 0.64	5.06 ± 0.70	0.783
Basophil (%)	0.18 ± 0.04	0.17 ± 0.05	0.21 ± 0.10	0.17 ± 0.12	0.944
Eosinophil (%)	1.83 ± 0.17	1.86 ± 0.21	1.79 ± 0.46	1.72 ± 0.31	0.959
Platelet count (x10^4^cells/mm^3^)	29.13 ± 0.58	28.53 ± 0.67	30.70 ± 1.63	30.70 ± 1.60	0.252
NLR	2.00 ± 0.06	1.95 ± 0.06	1.99 ± 0.12	2.33 ± 0.33	0.128
MLR	0.17 ± 0.01	0.17 ± 0.01	0.18 ± 0.02	0.17 ± 031	0.868
PLR	123.5 ± 3.4	127.1 ± 4.2	110.9 ± 6.6	117.7 ± 9.0	0.214

Data are shown as mean ± SEM. Analyses were performed using ANOVA. * Statistical significance was set at p < 0.05.

Multiple post-hoc comparisons were performed using Bonferroni test. ^a^ statistical difference between lean vs overweight; ^b^ statistical difference between lean vs obese.

MDD, major depressive disorder; n, number of patients; RBC, red blood cell; MCV, mean cell volume; MCH, mean corpuscular hemoglobin; MCHC, mean corpuscular hemoglobin concentration; RDW, red cell distribution width; WBC, white blood cell; NLR, neutrophil-to-lymphocyte ratio; MLR, monocyte-to-lymphocyte ratio; PLR, platelet-to-lymphocyte ratio; g/dL, grams per deciliter; fl, femtoliters; pg, picograms.

*Post-hoc* comparisons were performed using the Bonferroni test. In overweight MDD patients, the RBC, hematocrit, WBC, and neutrophil counts were elevated compared to lean MDD patients. While hemoglobin and monocyte count showed an upward trend in overweight MDD, the differences were not statistically significant. Obese patients with MDD had higher WBC and neutrophil counts than their lean counterparts. Moreover, lymphocyte counts and NLR showed an increasing trend in obese patients compared to lean MDD patients. Comparing overweight and obese patients with MDD, no significant differences in CBC parameters or inflammatory ratios were observed.

### 3.3 Linear relationship between BMI, 8Q score, 9Q score, complete blood parameters, and inflammatory ratios in MDD patients

The p -value and Pearson correlation coefficients (*r*) of the linear relationship between two continuous variables are summarized in **Table 3**. BMI had a weak positive correlation with the 8Q score, RBC count, hemoglobin, hematocrit, WBC count, neutrophil count, neutrophil percentage, monocyte count, plate count, and NLR (with correlation coefficients of 0.12, 0.22, 0.20, 0.22, 0.35, 0.34, 0.13, 0.20, 0.10, and 0.18, respectively), and a weak negative correlation with lymphocyte count, lymphocyte percentage, and PLR (with correlation coefficients of -0.20, -0.18, and -0.10, respectively). The 8Q score had a moderate correlation with the 9Q score (with a correlation coefficient of 0.63 and a p -value of < 0.0001). The 8Q score had a weak positive correlation with BMI, red cell distribution width (RDW), neutrophil count, neutrophil percentage, monocyte count, monocyte percentage, NLR, and MLR (with correlation coefficients of 0.12, 0.013, 0.13, 0.18, 0.15, 0.11, 0.15, and 0.20, respectively), and a weak negative correlation with lymphocyte percentage (with a correlation coefficient of -0.26 and a p -value of 0.019). The 9Q score had a weak correlation with lymphocyte count, monocyte percentage, eosinophil percentage, platelet count, and MLR (correlation coefficients of 0.10, 0.12, -0.10, -0.16, and 0.16, respectively).

**Table 3:** The relation between BMI, 8Q score, 9Q score, CBC parameters and inflammatory ratios in patients with MDD.

Parameters	BMI		8Q score		9Q score	
	*r*	P value	*r*	P -value	*r*	P -value
BMI	1		0.12*	0.186	0.09	0.305
8Q score	0.12*	0.186	1		0.63*	<0.0001*
9Q score	0.09	0.305	0.63*	<0.0001*	1	
RBC count	0.22*	0.010*	0.02	0.843	0.05	0.545
Hemoglobin	0.20*	0.021*	-0.06	0.524	0.09	0.330
Hematocrit	0.22*	0.012*	-0.07	0.435	0.05	0.547
MCV	-0.02	0.842	-0.07	0.392	0.02	0.858
MCH	-0.01	0.897	-0.06	0.466	0.04	0.618
MCHC	0.00	0.990	-0.05	0.602	0.07	0.402
RDW	-0.04	0.616	0.13*	0.121	0.06	0.503
WBC count	0.35*	<0.0001*	0.09	0.323	-0.03	0.723
Neutrophil count	0.34*	<0.0001*	0.13*	0.143	-0.01	0.954
Neutrophil (%)	0.13*	0.135	0.18*	0.036*	0.03	0.723
Lymphocyte count	-0.20*	0.020*	-0.07	0.405	-0.10*	0.269
Lymphocyte (%)	-0.18*	0.040*	-0.26*	0.002*	-0.08	0.332
Monocyte count	0.20*	0.023*	0.15*	0.093	0.08	0.361
Monocyte (%)	0.02	0.814	0.11*	0.197	0.12*	0.180
Basophil (%)	0.02	0.827	-0.06	0.458	-0.06	0.458
Eosinophil (%)	-0.00	0.964	-0.05	0.565	-0.10*	0.259
Platelet count	0.10*	0.246	0.01	0.933	-0.16*	0.072
NLR	0.18*	0.040*	0.15*	0.078	0.05	0.604
MLR	0.06	0.504	0.20*	0.019*	0.16*	0.065
PLR	-0.10*	0.262	0.06	0.473	-0.02	0.835

Correlation coefficients (r): 0.10 - 0.39 = weak correlation; 0.40 - 0.69 = moderate correlation; 0.70 – 0.89 = strong correlation; 0.90 – 1.00 = very strong correlation. Analyses were performed using bivariate analysis. *Correlation is significant at the 0.05 level (2-tailed). n = 135 cases. * Statistical significance was set at p < 0.05.

BMI, body mass index; r, correlation coefficients; 8Q score, 8 Questionnaire score; 9Q score, depression scale from PHQ-9 scale; MDD, major depressive disorder; RBC, red blood cell count; MCV, mean cell volume; MCH, mean corpuscular hemoglobin; MCHC, mean corpuscular hemoglobin concentration; RDW, red cell distribution width; WBC, white blood cell; NLR, neutrophil-to-lymphocyte ratio; MLR, monocyte-to-lymphocyte ratio; PLR, platelet-to-lymphocyte ratio.

### 3.4 Levels of serum inflammatory cytokines in different BMI groups in patients with MDD

**Table 4** summarizes levels of serum cytokines in patients with MDD with different BMI groups. Significant differences were observed in the levels of IL-1β and IL-6 among the BMI groups (p = 0.029 and 0.007, respectively). Levels of IFN-γ and TNF-α appeared to be higher in obese or overweight MDD patients compared to lean individuals, though the difference was not statistically significant (p = 0.135 and 0.061, respectively). TGF-β1 levels showed a decreasing trend with increasing body weight in patients with MDD, though this was not significant (p = 0.299).

**Table 4:** Level of serum cytokines in patients with MDD with different BMI groups.

Cytokines (pg/ml)	Lean MDD (*n* =98)	Overweight MDD (*n* =19)	Obese MDD (*n* =18)	P -value
IFN-γ	133.1 ± 12.4	188.5± 33.7	179.4 ± 37.6	0.135
IL-1β	32.7 ± 2.5	47.0 ± 5.5	46.3 ± 8.8	0.029*
IL-6	38.8 ± 5.4	85.0 ± 22.3	63.8 ± 11.9	0.007*
IL-8	141.9 ± 18.9	152.1 ± 51.2	157.5 ± 49.5	0.941
MCP-1	154.7 ± 11.4	149.0 ± 23.4	112.4 ± 21.9	0.324
TNF-α	33.9 ± 4.8	60.2 ± 17.3	57.4 ± 14.0	0.061
TGF- β1	45882 ± 5235	54479 ± 13770	28764 ± 9283	0.299

Data are shown as mean ± SEM. Analyses were performed using one-way ANOVA. * Statistical significance was set at p < 0.05.

MDD, major depressive disorder; SE, standard error of mean; 95% CI, 95% Confidence Interval for mean; IFN-γ, interferon-gamma; IL, interleukin; MCP-1, Monocyte Chemoattractant Protein-1; TNF-α, Tumor necrosis factor alpha; TGF- β1, Transforming growth factor-beta 1.

**Table 5** details the p-value of serum IL-1β and IL-6 levels in patients with MDD from various BMI groups as determined by *post-hoc* pairwise comparisons. Overweight patients with MDD exhibited significantly elevated levels of IL-1β and IL-6 compared to their lean counterparts (p = 0.036 and 0.003, respectively). Additional, obese patients with MDD had significantly higher IL-1β levels than lean patients with MDD (p = 0.005).

**Table 5:** Comparison of serum cytokines between BMI groups of patients with MDD.

Cytokines	P -value		
	Lean vs Overweight	Lean vs Obese	Overweight vs Obese
IL-1β	0.036*	0.050*	0.940
IL-6	0.003*	0.114	0.293

Analyses were performed using one-way ANOVA. * Statistical significance was set at p < 0.05.

MDD, major depressive disorder; SE, standard error of mean; IL, interleukin.

## 4 Discussion

Depression and obesity are now recognized as a state of chronic low-grade systemic inflammatory conditions. However, the impact of the coexistence of obesity and depression on the prognosis of depression and its precise mechanisms remain unclear. The present study aimed to determine whether coexist of overweight or obesity and depression increased the risk of having severe depression and a high risk of suicide. Additionally, this study investigated whether CBC parameters, inflammatory ratios, and proinflammatory cytokine production are influenced by the coexistence of obesity and depression in adolescents.

Comparing the odds of having high suicide risk and severe depression among different BMI groups of MDD patients, we found that overweight and obese MDD patients had higher odds of experiencing high suicide risk and severe depression compared to lean individuals. Our result is consistent with a previous study by Blasco et al. (2020) (26), who reported that the comorbidity of depression and obesity is a risk factor for poor prognosis.

Under lean conditions, adipose tissue is enriched in immune cells of type 2 immunity (predominantly anti-inflammatory/immune regulatory primed immune cells, such as M2-type macrophages, regulatory T cells (Treg), T-helper (Th)2, and type 2 innate lymphoid cells (ILC2). In obesity, the adipose immune system is dysregulated and replaced by inflammatory cells (for example, M1 macrophages, Th1, Th17, and CD8^+^ T cells) (13, 38) that secret proinflammatory cytokines such as IL-1β, IL-6, IL-17, and IFNγ (19– 21). Together, these changes lead to an elevated inflammatory state in obese individuals, with a predominance of neutrophils and hallmark increases in Th1 and Th17 cells (13, 19–21). This raises the possibility that the coexistence of two systemic inflammatory conditions (obesity and depression) will amplify the inflammatory responses and lead to worse outcomes and increased suicide risk in adolescents MDD patients.

To investigate the potential mechanisms that link the comorbidity of obesity and depression to the poor prognosis in these adolescents, we compared CBC parameters, inflammatory ratios, and levels of proinflammatory cytokines (IFN-γ, IL-1β, IL-6, IL-8, MCP-1, and TNF-α) and anti-inflammatory cytokine [TGF-β1]) between the different BMI groups. Here, we reported that WBC and neutrophil counts increased significantly with increasing body weight in patients with MDD. The lymphocyte percentage decreased with increasing body weight in patients with MDD, but this was not statistically different. Platelet count and NLR increased with increasing body weight in patients with MDD, but this was not statistically significant. When compared to lean patients with MDD, overweight patients with MDD had significantly higher RBC, WBC, neutrophil, and lymphocyte counts. The 8Q score, hemoglobin, hematocrit, and monocyte count tended to be higher in overweight MDD compared to lean individuals, but these were not statistically different. Obese patients with MDD had significantly higher WBC and neutrophil counts. The NLR tended to be higher in obese patients than in lean patients with MDD. In addition, the Pearson correlation coefficients (*r*) between the 8Q and 9Q scores, CBC parameters, and inflammatory ratios with BMI were analyzed. We reported that BMI had a weak positive correlation with the 8Q score, hemoglobin, hematocrit, RBC, WBC, neutrophil, monocyte, and plate counts, neutrophil percentage, and NLR, and a weak negative correlation with lymphocyte count, lymphocyte percentage, and PLR. The 8Q score had a weak positive correlation with BMI, neutrophil, and monocyte counts, neutrophil, and monocyte percentages, NLR, and MLR and a weak negative correlation with lymphocyte percentage.

There is emerging evidence supporting the association of immune system and pathophysiology of depression, with cytokines and chemokines standing front and center in this connection. Elevated levels of proinflammatory cytokines, including IL-1, IL-6, and TNF-α, have been consistently found in the blood of individuals experiencing MDD (5, 15, 22, 23), suggesting a heightened state of inflammation in these patients. Systemic inflammation can induce neuroinflammation by several mechanisms, including the break down of the blood-brain barrier, activation of glial cells associated with systemic immune activation, and effects on autonomic nerves via the organ-brain axis (39). Neuroinflammation, driven by these cytokines, can influence neurochemical imbalances, neurotransmitter function, and neuroendocrine activity and lead to neuron degeneration. This contributes to mood changes and the depressive symptoms seen in depression. Furthermore, these cytokines can disrupt the blood-brain barrier, promote oxidative stress, and stimulate the production of more inflammatory agents within the brain, creating a vicious cycle that may sustain or exacerbate depressive symptoms (39). In addition, a systematic review and meta-analysis, including 18 studies found that levels of IL-1β and IL-6 were significantly increased in blood and postmortem brain samples of patients with suicidality compared with both patients without suicidality and healthy control subjects (40). Contrary to proinflammatory cytokines, anti-inflammatory cytokines like IL-10 and TGF-β act to suppress inflammatory responses (38, 41). A decreased level of these cytokines may lead to unchecked inflammation, which is a consistent finding in many individuals with depression. As such, a balance between pro and anti-inflammatory cytokines is essential for mental well-being. Our results on cytokine levels in different BMI groups revealed that overweight and obese MDD patients had higher IL-1β and IL-6 levels than their lean individuals.Levels of IFN-γ and TNF-α also appeared to be higher in both overweight and obese patients when compared to lean MDD patients, while TGF-β1 levels appeared to decline as body weight increased. Collectively, our findings provide evidence that the coexistence of two low-grade systemic inflammatory conditions (obesity and depression) amplifies the inflammatory responses, consequently, leading to worse outcomes and increased suicide risk in adolescent MDD patients.

## 5 Conclusion

In adolescent MDD patients, the coexistence of overweight or obesity alongside depression amplifies the inflammatory status, which may contribute to a heightened severity of depression and a high suicide risk. In these patients, enhanced inflammation may be indicated by changes in CBC parameters including elevated levels of WBC, neutrophil, monocyte, and platelet counts, along with reduced lymphocyte percentages. This is further supported by increased inflammatory ratios (NLR and MLR), as well as elevated levels of the proinflammatory cytokines, specifically IL-1β and IL-6.

## 6 Limitations and strengths

This study has a limitation in that it was cross-sectional; thus, we cannot determine whether increasing total WBC, inflammatory immune cells (neutrophils, monocytes, platelets) lowered lymphocyte levels, and the expression of severe depressive symptoms in MDD followed or preceded obesity. Additionally, it is important to acknowledge that the physiological interactions between obesity, depression, and other environmental and endogenous factors are complex. Further studies on immune cell-adipocyte crosstalk, interactions of obese adipose tissues with high suicide risk and severe depressive symptoms, and mechanisms that link obese adipose tissue to poor prognosis of depression are required. The strengths of this study include the analysis of cytokines along with CBC with differentials and inflammatory ratios (NLR, MLR, and PLR) to figure out why adolescents with MDD who are overweight or obese are associated with higher suicide risk and severe depressive symptoms than lean individuals. Furthermore, we controlled for age, sex, and education level, as well as confounding factors or comorbidities that may affect inflammatory changes, such as other mood disorders, psychiatric disorders, immune system and hematological system disorders, infectious or inflammatory diseases, chronic physical illnesses, and other factors such as the use of alcohol, tobacco, antidepressants, and drugs that may affect inflammatory changes. Therefore, these variables did not affect the conclusions.

## 7 Implications

Our findings may have several implications. First, it raises the possibility that weight loss or reduced body weight could be a simple approach to lowering the risk of severe depressive symptoms and high suicide risk in MDD patients. Second, it is important to recognize that the impact of being overweight can be as profound as obesity itself in escalating the risk of severe depression and suicide in adolescents with MDD.

## Data availability statement

The original contributions presented in the study are included in the article/**Supplementary Material**. Further inquiries can be directed to the corresponding author.

## Ethics statement

The studies involving humans were approved by The Institutional Review Board of Walailak University. The studies were conducted in accordance with the local legislation and institutional requirements. The participants provided their written informed consent to participate in this study.

## Author contributions

PN, PP, and PK contributed to the conception, design of the study, and funding acquisition. PN, PP, and HJ collected data and performed the statistical analysis. PN wrote the first draft of the manuscript. PN, PP, PK, and KN wrote sections and revised the manuscript. All authors contributed to the manuscript revision, read, and approved the submitted version.
