# Supplementary material for: Being overweight and obese increases suicide risk, the severity of depression, and the inflammatory response in adolescents with major depressive disorders
Source: Front Immunol. 2023 Nov 1;14:1197775. doi: 10.3389/fimmu.2023.1197775 (PMC10646409; doi:10.3389/fimmu.2023.1197775)
Supplement: Supplementary file 1 [file DataSheet_1.docx]

**Supplementary Tables**

**Supplementary Table S1** Characteristics of **the participants.**

| Characteristics | All MDD  (*n*=135, 100%) | Lean MDD  (*n* =98, 72.6%) | Overweight MDD  (*n* =19, 14.1%) | Obese MDD (*n* =18, 13.3%) | P-value |
| --- | --- | --- | --- | --- | --- |
| BMI |  | - | - | - | <0.0001* |
| Median ± SD | 21.63 ± 6.08 | 20.42 ± 2.43^a, b^ | 26.88 ± 1.27^c^ | 33.84 ± 5.00 |  |
| Mean ± SEM | 23.12 ± 0.52 | 20.11 ± 0.24^a, b^ | 26.86 ± 0.29^c^ | 35.56 ± 1.18 |  |
| Sex, *n*(%) |  |  |  |  | 0.036* |
| Male | 41 (30.4)^d^ | 24 (24.5) | 10 (52.6) | 7 (38.9) |  |
| Female | 94 (69.6)^d^ | 76 (77.5) | 9 (47.4) | 11 (61.1) |  |
| Age (years) |  |  |  |  | 0.090 |
| Median ± SD | 20.0 ± 1.3 | 20.0 ± 1.3 | 21.0 ± 1.5 | 21.0 ± 1.4 |  |
| Mean ± SEM | 20.4 ± 0.1 | 20.3 ± 0.1 | 21.0 ± 0.3 | 20.7 ± 0.3 |  |
| Range (minimum to maximum) | 18 – 24 | 18 – 24 | 18 – 24 | 18 – 24 |  |
| State of depressive episode, *n*(%) |  |  |  |  | 0.852 |
| First episode | 123 (91.1) | 89 (90.8) | 17 (89.5) | 17 (94.4) |  |
| Recurrent depression | 12 (8.9) | 9 (9.2) | 2 (10.5) | 1 (5.6) |  |
| Smoking status, n(%) |  |  |  |  | 0.957 |
| Non smokers | 129 (95.56) | 94 (95.92) | 18 (94.74) | 17 (94.44) |  |
| Light smokers | 6 (4.44) | 4 (4.08) | 1 (5.26) | 1 (5.56) |  |
| Moderate -heavy smokers | 0 (0) | 0 (0) | 0 (0) | 0 (0) |  |

Analyses were performed using chi-squared tests for categorical data (sex, state of depressive episode, and smoking status), Kruskal-wallis or ANOVA test for continuous data (BMI, and age). Multiple post-hoc comparisons were performed for BMI. ^a^ statistical difference between lean vs overweight; ^b^ statistical difference between lean vs obese; ^c^ statistical difference between overweight vs obese. * Statistical significance was set at p < 0.05.

Abbreviations: MDD, major depressive disorder; *n*, number of patients; SD, standard deviation; SEM, standard error of mean; BMI, body mass index; Lean (< 25 kg/m^2^), overweight (25 to 29.9 kg/m^2^), and obese (≥ 30 kg/m^2^).

**Supplementary Table S2** 8Q score, 9Q score, depressive severity, and suicidal behaviour in patients with MDD with different BMI groups.

| Parameters | All MDD  (*n*=135, 100%) | Lean MDD  (*n* =98, 72.6%) | Overweight MDD  (*n* =19, 14.1%) | Obese MDD (*n* =18, 13.3%) | P-value |
| --- | --- | --- | --- | --- | --- |
| 8Q score |  |  |  |  | 0.158 |
| Mean ± SD | 7.7 ± 11.3 | 6.6 ± 10.7 | 11.5 ± 12.8 | 9.7 ± 11.8 |  |
| Mean ± SEM | 7.7 ± 1.0 | 6.6 ± 1.1 | 11.5 ± 2.9 | 9.7 ± 2.8 |  |
| 9Q score |  |  |  |  | 0.542 |
| Mean ± SD | 13.0 ± 5.3 | 12.7 ± 5.4 | 13.4 ± 5.2 | 14.1 ± 4.8 |  |
| Mean ± SEM | 13.0 ± 0.5 | 12.7 ± 0.6 | 13.4 ± 1.2 | 14.1 ± 1.1 |  |
| Suicide risk, n(%) |  |  |  |  | 0.188 |
| Low | 95 (70.3) | 73 (74.5) | 10 (52.6) | 12 (66.7) |  |
| Moderate | 16 (11.9) | 12 (12.2) | 3 (15.8) | 1 (2.1) |  |
| high | 24 (17.8) | 13 (13.3) | 6 (31.6) | 5 (27.8) |  |
| Depressive severity, n(%) |  |  |  |  | 0.325 |
| Mild | 86 (63.7) | 67 (68.4) | 9 (47.4) | 10 (55.6) |  |
| Moderate | 9 (6.7) | 6 (6.1) | 1 (5.3) | 2 (11.1) |  |
| Moderately severe | 3 (2.2) | 3 (3.1) | 0 (0.0) | 0 (0.0) |  |
| Severe | 37 (27.4) | 22 (22.4) | 9 (47.4) | 6 (33.3) |  |
| Types of suicidal behavior, n(%) |  |  |  |  | 0.427 |
| NSI | 57 (42.2) | 44 (44.9)) | 7 (36.8) | 6 (33.33) |  |
| SI | 36 (26.7) | 27 (27.55) | 3 (15.8) | 6 (33.33) |  |
| SA | 42 (31.1) | 27 (27.55) | 9 (47.4) | 6 (33.33) |  |

Analyses were performed using chi-squared tests for categorical data (depressive severity and types of suicidal behavior) and ANOVA for continuous data (8Q and 9Q score). * Statistical significance was set at p < 0.05.

Abbreviations: 8Q score, depression scale from 8Q scale; 9Q score, depression scale from PHQ-9 scale; MDD, major depressive disorder; *n*, number of patients; NSI, non-suicidal ideation; SI, suicidal ideation; SA, suicidal attempter. Based on the 8Q score, suicidality can be classified as no suicide risk (score = 0), low suicide risk (score = 1–8), moderate suicide risk (score = 9–16), or high suicide risk (score ≥ 17). PHQ-9 scores of 5, 10, 15, and 20 represent mild, moderate, moderately severe, and severe depression, respectively.
